# Reduced Functional Connectivity in Children With Congenital Cataracts Using Resting-State Electroencephalography Measurement

**DOI:** 10.3389/fnins.2021.657865

**Published:** 2021-04-14

**Authors:** Wan Chen, Liping Lan, Wei Xiao, Jiahong Li, Jiahao Liu, Fei Zhao, Chang-Dong Wang, Yiqing Zheng, Weirong Chen, Yuexin Cai

**Affiliations:** ^1^State Key Laboratory of Ophthalmology, Zhongshan Ophthalmic Center, Sun Yat-sen University, Guangzhou, China; ^2^Department of Otolaryngology, Sun Yat-sen Memorial Hospital, Sun Yat-sen University, Guangzhou, China; ^3^Institute of Hearing and Speech-Language Science, Sun Yat-sen University, Guangzhou, China; ^4^Department of Speech and Language Therapy and Hearing Science, Cardiff Metropolitan University, Cardiff, United Kingdom; ^5^Department of Hearing and Speech Science, Xinhua College, Sun Yat-sen University, Guangzhou, China; ^6^School of Data and Computer Science, Sun Yat-sen University, Guangzhou, China

**Keywords:** congenital cataracts, EEG, alpha-band, functional connectivity, attentive control

## Abstract

**Objectives:**

Numerous task-based functional magnetic resonance imaging studies indicate the presence of compensatory functional improvement in patients with congenital cataracts. However, there is neuroimaging evidence that shows decreased sensory perception or cognition information processing related to visual dysfunction, which favors a general loss hypothesis. This study explored the functional connectivity between visual and other networks in children with congenital cataracts using resting state electroencephalography.

**Methods:**

Twenty-one children with congenital cataracts (age: 8.02 ± 2.03 years) and thirty-five sex- and age-matched normal sighted controls were enrolled to investigate functional connectivity between the visual cortex and the default mode network, the salience network, and the cerebellum network during resting state electroencephalography (eyes closed) recordings.

**Result:**

The congenital cataract group was less active, than the control group, in the occipital, temporal, frontal and limbic lobes in the theta, alpha, beta1 and beta2 frequency bands. Additionally, there was reduced alpha-band connectivity between the visual and somatosensory cortices and between regions of the frontal and parietal cortices associated with cognitive and attentive control.

**Conclusion:**

The results indicate abnormalities in sensory, cognition, motion and execution functional connectivity across the developing brains of children with congenital cataracts when compared with normal controls. Reduced frontal alpha activity and alpha-band connectivity between the visual cortex and salience network might reflect attenuated inhibitory information flow, leading to higher attentional states, which could contribute to adaptation of environmental change in this group of patients.

## Introduction

Congenital cataracts are one of the most common causes of vision decline or blindness in children. Cloudy crystalline lenses interfere with light passage to the retina after birth leading to diverse etiologies (Lloyd et al., 1992). Previous neuroimaging studies have shown that visual deprivation can induce compensatory plasticity of the cortex. For example, task-based fMRI studies have shown that the occipital cortex of congenitally blind patients may process tactile (Dambrosia et al., 1997; Burton et al., 2006; Renier et al., 2010) and auditory information (Poirier et al., 2006; Chan et al., 2012; Föcker et al., 2012; Cappagli et al., 2017), and be involved in many high-level cognitive functions (Amedi et al., 2003; Raz et al., 2005; Burton and McLaren, 2006; Bedny et al., 2011, 2012, 2015). The processing of non-visual information by the occipital cortex, known as cross-modal reorganization, has been shown in studies using simultaneous audio-visual stimuli (Stein et al., 2010; Stein and Rowland, 2011). Neuroplasticity within occipital areas has been demonstrated to enhance auditory abilities in individuals with congenital blindness (Guerreiro et al., 2016). To explore intrinsic brain activity associated with memory in early blindness, a resting state fMRI study by Burton et al. (2014) showed increased functional connectivity between the visual cortex and prefrontal and parietal regions, i.e., the visual cortex exhibited a greater connection with the dorsolateral frontal cortex, the posterior intraparietal sulcal and the left fusiform gyrus in individuals with early blindness. These results support the view that after early visual deprivation the visual cortex may perform cognitive functions via enhanced functional connectivity with fronto-parietal control networks, which are dominant in memory and attention control.

In contrast, there is neuroimaging evidence showing reduced brain connectivity related to visual dysfunction. For example, an MRI study examined patients with bilateral cataracts and showed reduced brain function and structural connectivity in visual and cognitive-related brain areas, with significant improvements in functional and structural connectivity following cataract surgery (Lin et al., 2018). In addition, Liu et al. (2007) identified decreased functional connectivity within the occipital cortices and between the occipital cortices and the parietal somatosensory, frontal motor and temporal multisensory cortices in individuals with early blindness. Moreover, it was observed that children with amblyopia, one of the common results of congenital cataracts, had reduced functional connectivity between the default mode network, the salience network, and the primary visual cortex network (Dai et al., 2019). Reduced functional connectivity between the occipital and the posterior parietal cortices, the premotor cortex, the frontal insula and the dorsal prefrontal cortices has also been reported in children with amblyopia (Wang T. et al., 2014). A previous study demonstrated that cataract patients may have an accelerated age-related decline in brain function (Rogers and Langa, 2010). This evidence favors a general loss hypothesis that refers to maladaptive plasticity due to early visual loss, such as a decreased ability in processing sensory perception or spatial information (Zwiers et al., 2001; Amedi et al., 2005; Pascual-Leone et al., 2005). It is hypothesized that early visual loss probably prevents the development of the occipital cortex and the establishment of functional connectivity between visual regions and other brain regions (Liu et al., 2007). However, the general loss hypothesis has not been studied widely using task-based fMRI, because visual tasks cannot be performed on patients with congenital cataracts in most cases.

Without the requirement to view a stimulus or give a response, resting state electroencephalography (rsEEG) could be an effective tool to investigate spontaneous neuronal activity and connectivity across the brain at a given frequency. In previous studies, synchronization and coherence of source signals using rsEEG have been conducted to identify functional connectivity between different brain regions (van Diessen et al., 2015). Several studies (Damoiseaux et al., 2006; De Luca et al., 2006) found reproducible patterns with potential functional relevance by applying probabilistic independent component analysis. These results suggest that the brain is organized into multiple brain networks, and thus studies on network connectivity have shed light on the large-scale functional organization of abnormal brains (Buckner and Vincent, 2007; Fox and Raichle, 2007). The study of network connections using rsEEG data may provide insight into brain plasticity associated with congenital cataracts.

It is accepted that visual deprivation before and after sensitive periods may lead to different functional connectivity patterns within the occipital cortex (Buchel et al., 1998; Collignon et al., 2013). A task-based study by Collignon et al. showed that there were stronger connections between the primary visual cortex and the primary auditory cortex in response to auditory stimulation in congenitally blind individuals, whereas the primary visual cortex was closely related to parietal regions in late-onset blind individuals (Collignon et al., 2013). Thus, the underlying neural effects of congenital cataracts might be more comprehensive than reported previously as there may be additional neural functional connectivity across the brain.

In this study, the primary aim was to explore functional connectivity between the visual and other networks in children with congenital cataracts by using rsEEG. Regions of interest were identified from a recent study on amblyopia (Dai et al., 2019). Significant results could provide an important tool for identifying abnormalities in sensory, cognition, motion and execution functional connectivity across the brains in these children.

## Materials and Methods

### Participants

In consideration of the primary aim of the study and the sensitive visual deprivation period, twenty-one right-handed children with congenital cataracts (age: 8.02 ± 2.03 years, male: female = 14:7) and thirty-five age- and sex-matched (age: 9.09 ± 1.49 years, male: female = 5:2) right-handed normal sighted children were recruited by an ophthalmologist from the congenital cataracts department at Zhongshan Ophthalmic Center. All patients received a series of ocular examinations including visual acuity, intraocular pressure, refraction, ocular motility and slit lamp tests. The control subjects had normal or adjusted to normal visual acuity in both eyes, and had no history of ocular diseases, neurological disorders or brain abnormalities. Written informed consent was obtained from all children enrolled in the study or their legal guardians in accordance with the Declaration of Helsinki and the study was approved by the Ethics Committee of Zhongshan Ophthalmic Center, Sun Yat-sen University.

### EEG Data Collection

A high-density EEG with 128 channels (EGI, Eugene) and a NetAmps 200 amplifier was used to collect the rsEEG data of all participants for approximately 7 min. All subjects sat in an acoustic and electrically shielded and dark room and were instructed to close their eyes. They were also instructed to stay awake and move their head as little as possible. The CZ electrode formed the reference. The sampling rate was 1,000 Hz and electrode impedance kept below 20 kΩ.

### Preprocessing of EEG Data

EEGLAB for v13.0.0 toolbox in MATLAB for R2013a was used to preprocess the raw EEG data. The data were re-referenced against the mean reference for all electrodes at first. Then, the sampling rate was set to 0.5 kHz. A Sag filter was used to eliminate 50-Hz frequency interference and then a 0.1-Hz high-pass filter and a 100-Hz low-pass filter were applied. The reference electrodes were the bilateral mastoids. Gross artifacts were visually detected and manually removed. Then, artifacts originating from a limited volume of space or a few distinct sources were removed. Independent component analysis (ICA) was applied to remove eye movement, heartbeats and muscle artifacts (Delorme and Makeig, 2004). In addition, other artifacts such as exponential decay were also detected using ICA and subsequently removed. Then, traces were segmented in 2s epochs, and spectra of each epoch were calculated by fast Fourier transform (FFT), with a frequency resolution of 0.5 Hz, tapered by a Hanning window. Finally, the average Fourier cross-spectral matrices were calculated, including delta (2–3.5 Hz), theta (4–7.5 Hz), alpha1 (8–10 Hz), alpha2 (10–12 Hz), beta1 (13–18 Hz), beta2 (18.5–21 Hz), beta3 (21.5–30 Hz), gamma 1 (30.5–44 Hz) and gamma 2 (55–100 Hz).

### Data Analysis

#### Source Localization

Standardized low-resolution brain electromagnetic tomography (sLORETA) was used to explore the abnormal activity of brain areas in children with congenital cataracts. sLORETA was used to calculate neuronal activity as the current density (A/m^2^). LORETA-Key software was used to investigate the solution space and the lead field matrix. Applying the boundary element on the MMI-152, the software re-assessed realistic electrode coordinates (Jurcak et al., 2007) and the lead field (Fuchs et al., 2002). The sLORETA-Key anatomical template divided the MNI-152 volume into 6239 voxels according to probabilities returned by the Demon Atlas (Lancaster et al., 2015). The correct translation was used by co-registration from the MNI-152 space into the Talairach and Tournoux spaces.

#### Functional Connectivity

The amount of cross-talk between brain regions contributing to the activity of the source regions was used to interpret the lagged phase coherence between two brain areas. Since the two brain areas oscillate coherently with a phase lag, cross-talk can be interpreted as information sharing transmitted through axons. Using the discrete Fourier transform, the data were decomposed into a limited number of cosine and sine waves at Fourier frequencies. Pascual-Marqui showed that based on asymptotic results, the threshold is significant for a given lagged phase coherence value. sLORETA was applied to extract the time-varying current density for regions of interest (ROIs). Power in all voxels was normalized to a power of 1 and log transformed at each time point. Therefore, ROI values reflect the log transformed fraction of total power of all voxels for specific frequencies. The ROI was confirmed according to a previous study (Dai et al., 2019), as shown in Table 1.

**TABLE 1:** The regions of interest in each network.

Network	Regions of interest	BA	Peak MNI coordinates (mm)			z-scores
			
			X	Y	Z	
SN	rFIC	47	38	20	1	14.32
	lFIC	47	−33	19	−1	12.21
	ACC	24	−2.5	11.5	38.5	14.48
CEN	rDPC	9	48	22	45	15.64
	lDPC	9	−43.5	23.5	41.5	12.35
	rPPC	40	56	−50	43	14.87
	lPPC	40	−43.5	−52.5	49.5	11.41
DMN	VPC	11	0	−48	−15	14.08
	PCC	23/30	−6	−49	29	13.36
PVCN	rCAL	17	17.5	−99.5	4.5	12.1
	lCAL	17	−17.5	−99.5	4.5	13.65
HVCN	rLING	19	13	−55	−2	14.54
	rFFG	19	41	−62	−18	13.54
	rFFG	37	30	−47	−12	10.96
	lFFG	37	−30	−47	−12	12.05

*BA, Brodmann’s area; MNI, Montreal neurological institute; SN, salience network; CEN, cerebellum network; DMN, default mode network; PVCN, primary visual cortex network; HVCN, higher visual cortex network; rFIC, right frontoinsular cortex; lFIC, left frontoinsular cortex; ACC, anterior cingulate cortex; rDPC, right dorsolateral prefrontal cortex; lDPC, left dorsolateral prefrontal cortex; rPPC, right posterior parietal cortex; lPPC, left posterior parietal cortex; VPC, ventromedial prefrontal cortex; PCC, posterior cingulate cortex; rCAL, right calcarine cortex; lCAL, left calcarine cortex; rLING, right lingual gyrus; rFFG, fusiform gyrus; lFFC, fusiform gyrus.*

### Statistical Analysis

Non-parametric statistical analysis of the LORETA-KEY images (statistical non-parametric mapping, SnPM) was carried out to compare the differences in resting-state EEG activity between the children with congenital cataracts and normal sighted controls, using LORETA-KEY’s built-in voxel-wise randomization tests (5000 permutations). A *t* statistic test was used for independent groups with a threshold of *p* < 0.01 (corrected for multiple comparisons).

In SnPM, the correction for multiple comparisons using random permutation has been shown to yield results similar to those obtained from a statistical parametric mapping approach using a general linear model with multiple comparisons corrections. The *t*-statistic for independent groups was used to compare lagged connectivity differences between the two groups and *p* values less than 0.05 (two-sided) were considered statistically significant. LORETA-KEY’s built-in voxel-wise randomization tests were implied to correct for multiple comparisons, for all voxels included in the ROI for connectivity analysis (5000 permutations).

## Results

### Characteristics of Patients With Congenital Cataracts and Healthy Controls

Twenty-one children with congenital cataracts and thirty-five healthy controls were recruited for this study. No significant differences were found between the congenital cataracts group and the control group in terms of age (F = 1.365, *p* = 0.178) or sex (x^2^ = 0.141, *p* = 0.708). Table 2 shows congenital cataract eyes, best-corrected visual acuity, age of onset and light perception of patients with congenital cataracts.

**TABLE 2:** Demographic information of congenital cataracts subjects.

Subjects	Gender	Age (years)	Congenital cataracts eye	Best-corrected visual acuity (logMAR)		Light perception	Age of onset
				
				Right	Left		
CC 01	Male	4.5	Binoculus	0.30	0.30	Weak	0
CC 02	Female	11.5	Binoculus	0.60	0.60	Weak	0
CC 03	Male	8	Binoculus	0.49	0.49	Weak	0
CC 04	Female	13	Binoculus	1.70	1.70	Weak	0
CC 05	Male	9	Binoculus	0.49	0.49	Weak	0
CC 06	Male	7	Binoculus	1.52	1.52	Weak	0
CC 07	Female	9	Binoculus	0.22	0.22	Weak	0
CC 08	Female	6.5	Binoculus	0.52	0.52	Weak	0
CC 09	Male	9	Binoculus	0.52	0.52	Weak	0
CC 10	Male	5	Binoculus	0.70	0.70	Weak	0
CC 11	Male	6.5	Binoculus	0.80	0.80	Weak	0
CC 12	Male	11.5	Left eye	0.00	1.22	Weak	0
CC 13	Female	15	Left eye	0.00	0.52	Weak	0
CC 14	Female	8	Left eye	0.00	0.22	Weak	0
CC 15	Female	5	Left eye	0.00	1.00	Weak	0
CC 16	Male	10	Right eye	0.52	0.00	Weak	0
CC 17	Male	7	Right eye	0.40	0.00	Weak	0
CC 18	Male	4	Right eye	0.82	0.00	Weak	0
CC 19	Male	7	Right eye	0.30	0.00	Weak	0
CC 20	Male	9	Right eye	0.40	0.00	Weak	0
CC 21	Male	7	Right eye	0.52	0.00	Weak	0

### Brain Activity in Individuals With Congenital Cataracts in Contrast to Healthy Controls

**FIGURE 1:**
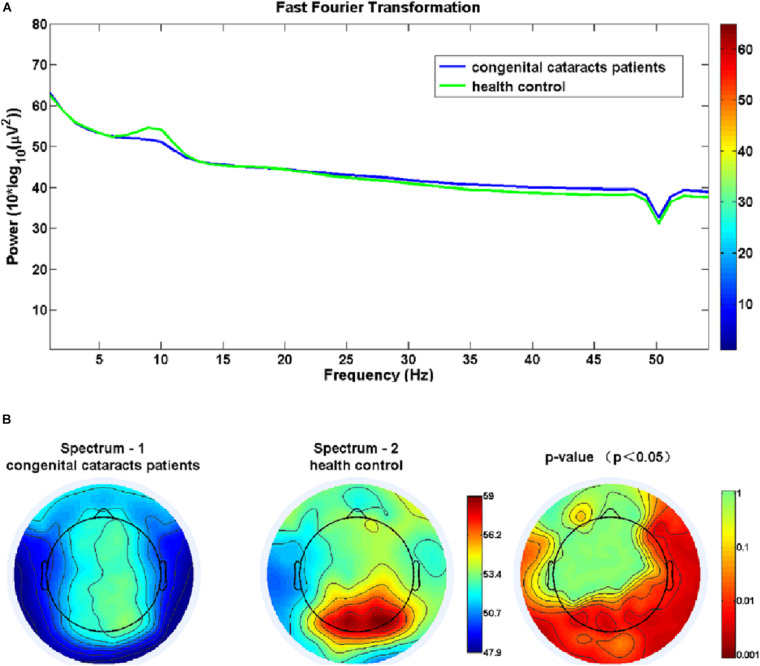
**(A)** Congenital cataract patients had a significant reduction in alpha frequency compared with healthy controls using fast Fourier transformation. **(B)** Significant group differences in the alpha frequency in the occipital and right temporal cortices (*p* < 0.05).

**FIGURE 2:**
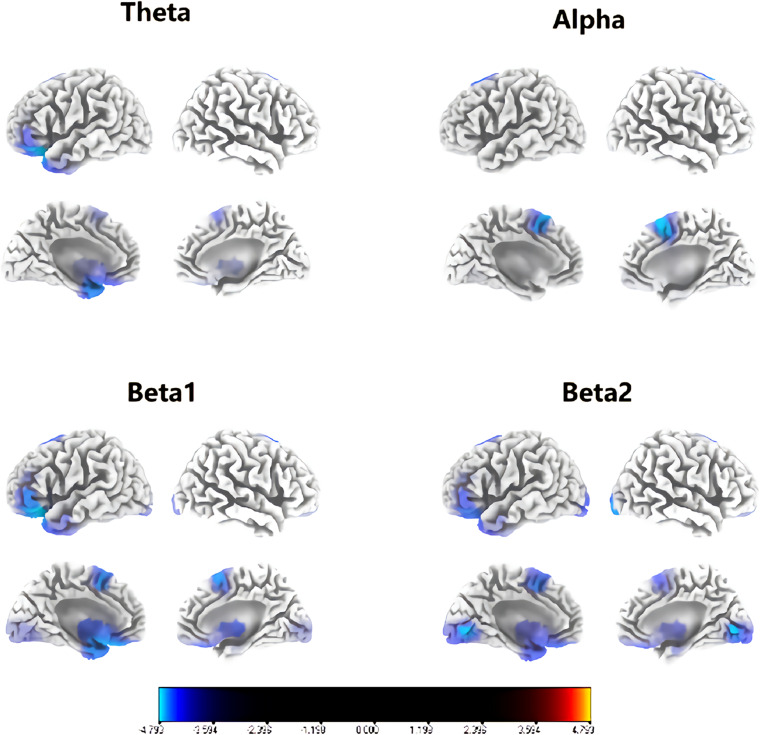
Regions with significant group differences for each frequency in sLORETA analysis (*p* < 0.05). Blue regions imply that the activity of the congenital cataract group was significantly lower than that of healthy controls.

Figure 1 shows that congenital cataract patients had a significant reduction in alpha frequency compared with healthy subjects with fast Fourier transformation (a). Additionally, there was decreased alpha activity distributed in the occipital cortex and the right temporal cortex (b). In the sLORETA analysis, shown in Figure 2, the inferior frontal gyrus (BA 47), the middle frontal gyrus (BA 11) and the extranuclear region (BA 13) showed significantly reduced activation in the theta band (*t* = −4.75, *p* < 0.01). Moreover, there was decreasing alpha activity in the superior frontal gyrus (BA 6), the middle frontal gyrus (BA 6) and the cingulate gyrus (BA 32) (*t* = –4.75, *p* < 0.01). Decreasing beta1 activity was distributed in the inferior frontal gyrus (BA 47), the rectal gyrus (BA 11), the orbital gyrus (BA 47), the superior temporal gyrus (BA 38), the extranuclear gyrus (BA 13), and the uncus gyrus (BA 34) (*t* = –3.85, *p* < 0.01). Decreasing beta2 activity was exhibited in the lingual gyrus (BA 17, 18) and the superior frontal gyrus (BA 6) (*t* = –3.875, *p* < 0.05).

### Comparison of FC Between ROIs in Individuals With Congenital Cataracts and Healthy Controls

Fifteen regions of interest (ROIs) were estimated by the ICA, involving five rest state networks (Table 1). The salience network (SN) includes the bilateral frontoinsular cortex and the anterior cingulate cortex (rFIC/lFIC/ACC). The cerebellum network (CEN) is composed of the bilateral dorsolateral prefrontal cortex and the posterior parietal cortex (rDPC/lDPC/rPPC/lPPC). The default mode network (DMN) consists of the ventromedial prefrontal cortex and the posterior cingulate cortex (VPC/PCC). Both bilateral calcarine cortices (rCAL/lCAL) are key nodes of the primary visual cortex network (PVCN), while the right lingual gyrus and the bilateral fusiform gyrus (rLING/rFFG/lFFG) form the higher visual cortex network (HVCN) Dai et al. (2019).

**FIGURE 3:**
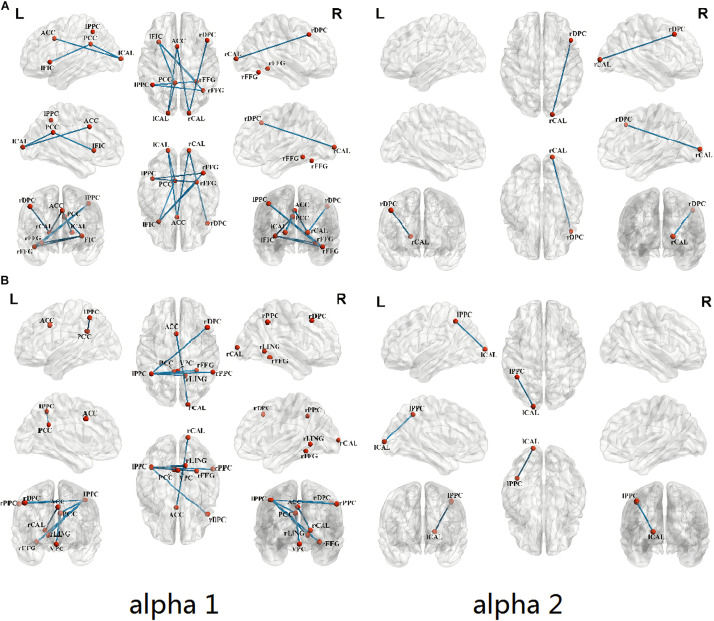
Comparison of FC between ROIs in children with congenital cataracts when compared with healthy controls (*p* < 0.05). **(A)** Compared with healthy controls, the congenital cataracts group showed a weak lagged coherence for alpha frequency between ROIs. **(B)** Significantly decreased lagged phase synchronization in congenital cataract patients in contrast to healthy controls.

Figure 3A shows that when compared with healthy controls, the congenital cataracts group showed a weak lagged coherence between the following ROIs: left frontoinsular cortex-right fusiform gyrus (SN-HVCN), anterior cingulate cortex-the bilateral calcarine cortex (SN-PVCN), right dorsolateral prefrontal cortex-right calcarine cortex (CEN-PVCN), left posterior parietal cortex-right fusiform gyrus (CEN-HVCN), posterior cingulate cortex-left frontoinsular cortex, left calcarine cortex (DMN-PVCN,SN), left calcarine cortex-anterior cingulate cortex, posterior cingulate cortex (SN,DMN-PVCN), right fusiform gyrus-left frontoinsular cortex, and left posterior parietal cortex (SN,CEN-HVCN).

In addition, as shown in Figure 3B, using lagged phase synchronization analysis there were significantly decreased functional connections in the congenital cataract patients between the following ROIs: anterior cingulate cortex-right calcarine cortex (SN-PVCN), posterior cingulate cortex-left posterior parietal cortex, ventromedial prefrontal cortex (DMN-CEN,DMN), left posterior parietal cortex-right dorsolateral prefrontal cortex, right posterior parietal cortex, posterior cingulate cortex, right lingual gyrus, right fusiform gyrus (CEN-CEN,DMN,HVCN), and left calcarine cortex-left posterior parietal cortex (CEN-PVCN). Relevant numerical data of values and stats on reported changes are included in the Supplementary Table.

## Discussion

The study innovatively investigated brain alterations in children between 6 and 14 years of age with congenital cataracts. In this study, rsEEG was recorded while subjects were asked to close their eyes and rest. Brain function was compared in congenital cataract and normal-sighted children in a multiple-level analysis. EEG measures of functional and directed coherence are supposed to be sensitive to different brain maturations (Michels et al., 2013). Thus, we speculated that early blindness-induced brain reorganization is possibly different from that in adults.

The results showed that the congenital cataract group had (1) decreased brain activity mainly in the occipital, temporal, frontal and limbic lobes and (2) reduced alpha-band connectivity between the visual and somatosensory cortices and between regions of the frontal and parietal cortices associated with cognitive and attentive control. These findings could support the general loss hypothesis, in that an absence of early visual input is likely to prevent the development of the occipital cortex and the establishment of functional connectivity between visual regions and other brain regions via synaptic revision (Liu et al., 2007).

It is noteworthy that children with congenital cataracts had a significant reduction in the alpha frequency, mostly in the occipital cortex and the right temporal cortex when compared with healthy subjects. This is in accordance with a task-based EEG study that showed reduced posterior alpha oscillations in subjects with a history of total vision loss after birth, although they had their sight restored after 4 months (Bottari et al., 2016). This proved that a lack of visual input during a sensitive phase in development may impair the generation of alpha oscillations. Considering that posterior alpha activity does not seem to emerge before late childhood (Anderson and Perone, 2018) and most likely results from pyramidal cells depending on GABA-mediated inhibition (Buffalo et al., 2011; Jensen et al., 2012), functional brain development might be experience-dependent and thus can be affected by an absence of early visual input.

sLORETA analysis revealed that in comparison to healthy controls, children with congenital cataracts showed decreased brain activity mainly in the frontal, occipital and limbic lobes. These regions are crucial in the visual pathway. The frontal lobe plays a critical role in visual information perception, memory, and regulation (Majaj et al., 2007). The occipital cortex mainly performs the functions of visual and motion perception. Damage to the occipital cortex may cause partial or complete blindness (Grill-Spector et al., 1998). The temporal cortex is involved in visual perception, facial recognition, memory association and formation (Shrager et al., 2006), and the limbic lobe is typically engaged in emotional and cognitive processing and associational integration (Vogt, 2005). Lower activity in these regions may indicate visual dysfunction in these patients. This is in accordance with other studies of abnormal visual decline, which showed lower local activity in the bilateral frontal, temporal, and occipital lobes (Dai et al., 2019).

ROI-FC was used to analyze whether congenital cataracts altered brain functional networks. The results showed decreased lagged coherence in the alpha frequency band between the PVCN and the SN, the DMN, and the CEN. The altered functional connection with the HVCN was similar to that with the PVC. In addition, decreased lagged phase synchronization was seen within the CEN and between the CEN and the DMN, the HVCN, and the PVCN. Thus, congenital cataracts might alter the alpha-band connections of the PVCN, the HVCN, the SN, the DMN and the CEN.

Remarkably, both interconnection in the CEN and connectivity between the left posterior parietal cortex of the CEN and the visual cortex were reduced for the alpha frequency band. The posterior parietal cortex, also known as the somatosensory association cortex, traditionally executes visual perception and spatial orientation functions. There is also accumulating evidence indicating its extensive involvement in cognitive functions (Akrami et al., 2018; Freedman and Ibos, 2018; Sestieri et al., 2017). The posterior parietal cortex between the visual cortex and the somatosensory cortex is strongly connected with the frontal lobe which is involved in complex cognitive functions including motor planning and working memory (Rowe et al., 2001; Levine et al., 2011). Therefore, decreased connection with the posterior parietal cortex implies visual deprivation induced spatial orientation dysfunction and impaired working execution such as motor planning. This is consistent with studies on congenital blindness (Liu et al., 2007; Yu et al., 2008; Burton et al., 2014; Wang D. et al., 2014), which confirmed a decreased FC between the visual cortex and the sensorimotor network.

Furthermore, visual cortex networks showed weak alpha-band connectivity with the posterior cingulate cortex of the DMN, the left frontoinsular cortex of the SN and the right dorsolateral prefrontal cortex of the CEN. Similarly, the alpha activity levels in both the cingulate gyrus and the frontal cortex were seen to decrease in this study. The posterior cingulate cortex, a key node of the DMN, is involved in attention and cognition associated integration (Leech and Sharp, 2014). The frontoinsular cortex is considered an information integration hub of the SN (Barnes et al., 2001; Li et al., 2011). The ventral stream passes through the visual cortex to the frontoinsular cortex and along the dorsal visual stream, with visual information transmitted from occipital to parietal and frontal regions. Additionally, the dorsolateral prefrontal cortex is mainly associated with recognition memory and attention control (McDermott et al., 2000; Gold and Buckner, 2002).

A top-down mechanism of alpha oscillations has been suggested to modulate brain attentional states, which has an important role in cognitive functions by enabling communication in neonates (Saalmann et al., 2012; Lopes Da Silva, 2013). It has been claimed that the dynamic cortico-thalamo-cortical circuit, which consists of connected areas involving GABAergic cells could account for the generation of alpha oscillations (Lopes Da Silva, 2013). Thus, information processing within a local neuronal region is activated with disinhibition by decreased alpha power. Decreased alpha activity has also been linked to the presence of attention. This is exemplified by a task-based study that showed lower alpha activity in response to an increased state of alertness (Del Zotto et al., 2013), generally referred to as event-related desynchronization. Alpha phase synchronization and long-distance phase coherence have also been consistently linked to attentive and cognitive control as a top-down modulation (Fries, 2005; Klimesch et al., 2006; Thangavel et al., 2011; Tipura et al., 2017). Thus, via suppression of irrelevant information, this top-down modulated mechanism could increase the signal-to-noise ratio in attentive and cognitive processing (Jensen and Mazaheri, 2010; Klimesch, 2012). In blind individuals, research has shown reduced alpha power in parieto-occipital electrodes in both sensory and cognitive tasks (Kriegseis et al., 2006).

It is speculated that the observed lower alpha activity in the frontal cortex might indicate that early visual deprivation induced a decrease in the number of inhibitory neurons for efficient attentive and cognitive processing. There is some evidence that brain activity measured by rsEEG is linked to brain networks. For example, Bonnard et al. (2016) found that alpha activity in the occipital region is associated with DMN activity. Consequently, reduced alpha-band connectivity between frontal and occipital areas might reflect an attenuated inhibitory information flow, leading to higher attentional states than in the sighted controls, which contributes substantially to the adaptation to environmental change in congenital cataracts. This is also in line with an fMRI study indicating impaired top-down modulation after visual deprivation in critical development periods (Wen et al., 2018). Another fMRI study showed decreased functional connectivity between the occipital and frontal lobes in congenital cataracts (Liu et al., 2007). This is likely to indicate reduced attention control in visual perception and memory due to visual deprivation in children with congenital cataracts.

There are some limitations in this study. This is a cross-sectional study with a limited sample size. Longitudinal studies are needed to further explore brain alterations in children with congenital cataracts. In addition, congenital cataract patients were heterogeneous in terms of differences in laterality. It was difficult to eliminate the effects of laterality completely with a limited sample size. Therefore, we should explain the results with caution. Furthermore, it would be interesting to explore the influence of various factors on central neural alterations in patients with congenital cataracts by categorizing them on the basis of different characteristics (e.g., cognitive performance, laterality of cataracts).

## Conclusion

This study has shown decreased activity and connectivity in the occipital, temporal, frontal and limbic lobes in children with congenital cataracts, which may support the general loss hypothesis. The reduced alpha-band connectivity between the visual cortex and regions of the frontal and parietal cortices associated with cognitive and attentive control might reflect an attenuated inhibitory information flow, leading to higher attentional states. This study contributes significantly to the adaptation to environmental change seen in children with congenital cataracts.

## Data Availability Statement

The raw data supporting the conclusions of this article will be made available by the authors, without undue reservation.

## Ethics Statement

The studies involving human participants were reviewed and approved by the Ethics Committee of Zhong Shan Ophthalmic Center, Sun Yat-sen University. Written informed consent to participate in this study was provided by the participants’ legal guardian/next of kin.

## Author Contributions

YC and WaC: conception and study design. LL and JLi: data collection. C-DW, JLiu, and WX: statistical analysis. WaC and LL: interpretation of results. WaC, LL, FZ, YZ, YC, and WeC: drafting the manuscript work or revising it critically for important intellectual content. All authors: approval of the final version to be published and agreement to be accountable for the integrity and accuracy of all aspects of the work.

## Conflict of Interest

The authors declare that the research was conducted in the absence of any commercial or financial relationships that could be construed as a potential conflict of interest.

## Abbreviations

SN: salience network
CEN: cerebellum network
DMN: default mode network
PVCN: primary visual cortex network
HVCN: higher visual cortex network
rFIC: right frontoinsular cortex
lFIC: left frontoinsular cortex
ACC: anterior cingulate cortex
rDPC: right dorsolateral prefrontal cortex
lDPC: left dorsolateral prefrontal cortex
rPPC: right posterior parietal cortex
lPPC: left posterior parietal cortex
VPC: ventromedial prefrontal cortex
PCC: posterior cingulate cortex
rCAL: right calcarine cortex
lCAL: left calcarine cortex
rLING: right lingual gyrus
rFFG: fusiform gyrus
lFFC: fusiform gyrus
rsEEG: resting state electroencephalography
fMRI: functional magnetic resonance imaging.
