# Supplementary material for: Reduced Functional Connectivity in Children With Congenital Cataracts Using Resting-State Electroencephalography Measurement
Source: Front Neurosci. 2021 Apr 14;15:657865. doi: 10.3389/fnins.2021.657865 (PMC8079630; doi:10.3389/fnins.2021.657865)
Supplement: Supplementary file 1 [file Table_1.DOCX]

| **Lagged coherence** | | | | | |
| --- | --- | --- | --- | --- | --- |
|  | Reduced connectivity between ROIs | | | *t* | *P* |
| **Figure 3. (a)**  **Alpha1 band** | IFIC | ←→ | PCC  rFFG | -3.8974 | ＜0.05 |
|  |  |  |  | -4.4591 | ＜0.01 |
|  | ACC | ←→ | rCAL  lCAL | -3.8780 | ＜0.05 |
|  |  |  |  | -3.8806 | ＜0.05 |
|  | rDPC | ←→ | rCAL | -4.0970 | ＜0.05 |
|  | IPPC | ←→ | rFFG  rFFG | -4.0577 | ＜0.05 |
|  |  |  |  | -4.2462 | ＜0.05 |
|  | PCC | ←→ | lCAL | -4.2110 | ＜0.05 |
| **Figure 3. (b)**  **Alpha2 band** | rDPC | ←→ | rCAL | -4.7201 | ＜0.05 |
| *t*＜-3.864，*p*＜0.05；*t*＜-4.441，*p*＜0.01 | | | | | |
|  | | | | | |
| **Lagged phase synchronization** | | | | | |
|  | Reduced connectivity between ROIs | | | *t* | *P* |
| **Figure 3. (a)**  **Alpha1 band** | ACC | ←→ | rCAL | -4.3147 | ＜0.01 |
|  | rDPC | ←→ | IPPC | -4.4950 | ＜0.01 |
|  | rPPC | ←→ | IPPC | -4.3347 | ＜0.01 |
|  | IPPC | ←→ | PCC  rFFG  rLING | -4.2247  -3.7247  -4.4863 | ＜0.05 |
|  |  |  |  |  | ＜0.05 |
|  |  |  |  |  | ＜0.01 |
|  | VPC | ←→ | PCC | -4.0314 | ＜0.05 |
| **Figure 3. (b)**  **Alpha2 band** | lCAL | ←→ | lPPC | -4.1541 | ＜0.05 |
| *t*＜-3.706，*p*＜0.05；*t*＜-4.305，*p*＜0.01 | | | | | |

Supplementary table1
